# Inhaled Pulmonary Vasodilators for the Treatment of Right Ventricular Failure in Cardio-Thoracic Surgery: Is One Better than the Others?

**DOI:** 10.3390/jcm13020564

**Published:** 2024-01-18

**Authors:** Maria Benedetto, Giulia Piccone, Leonardo Gottin, Andrea Castelli, Massimo Baiocchi

**Affiliations:** 1 Cardio-Thoracic and Vascular Anesthesia and Intensive Care Unit, IRCCS Azienda Ospedaliero-Universitaria di Bologna, Via Albertoni 15, 40138 Bologna, Italy; andrea.castelli@aosp.bo.it (A.C.); massimo.baiocchi@aosp.bo.it (M.B.); 2 Cardiothoracic and Vascular Intensive Care Unit, Hospital and University Trust of Verona, P. le A. Stefani, 37124 Verona, Italy; giulia.piccone@gmail.com (G.P.); leonardo.gottin@univr.it (L.G.)

**Keywords:** pulmonary vascular dysfunction, right ventricular failure, pulmonary hypertension, inhaled nitric oxide, inhaled prostacyclins, heart transplant, lung transplant, assist devices

## Abstract

Right ventricular failure (RFV) is a potential complication following cardio-thoracic surgery, with an incidence ranging from 0.1% to 30%. The increase in pulmonary vascular resistance (PVR) is one of the main triggers of perioperative RVF. Inhaled pulmonary vasodilators (IPVs) can reduce PVR and improve right ventricular function with minimal systemic effects. This narrative review aims to assess the efficacy of inhaled nitric oxide and inhaled prostacyclins for the treatment of perioperative RVF. The literature, although statistically limited, supports the clinical similarity between them. However, it failed to demonstrate a clear benefit from the pre-emptive use of inhaled nitric oxide in patients undergoing left ventricular assist device implantation or early administration during heart-lung transplants. Additional concerns are related to cost safety and IPV use in pathologies associated with pulmonary venous congestion. The largest ongoing randomized controlled trial on adults (INSPIRE-FLO) is addressing whether inhaled Epoprostenol and inhaled nitric oxide are similar in preventing RVF after heart transplants and left ventricular assist device placement, and whether they are similar in preventing primary graft dysfunction after lung transplants. The preliminary analysis supports their equivalence. Several key points may be achieved by the present narrative review. When RVF occurs in the setting of elevated PVR, IPV should be the preferred initial treatment and they should be preventively used in patients at high risk of postoperative RVF. If severe refractory postoperative RVF occurs, IPVs should be combined with complementary pharmacology (inotropes and inodilators). If unsuccessful, right ventricular mechanical support should be established.

## 1. Background

Right ventricular failure (RVF) is a potential complication following cardio-thoracic surgery, with an incidence ranging from 0.1% to 30%, depending on the surgical scenario. More specifically, the occurrences of perioperative RVF are 60% after mitral valve surgery, 20–30% after left ventricular assist device (LVAD) implantation, and 18% after heart transplantation (HT) [1,2,3].

This condition is associated with prolonged lengths of mechanical ventilation (MV) and an increased need for mechanical circulatory support (MCS) [4].

The increase in pulmonary vascular resistance (PVR) is one of the mechanisms that explain perioperative RVF. The pulmonary circulation is a high-flow, low-pressure system. However, several factors during cardiothoracic surgery can trigger an intrapulmonary vascular tone increase with increased right ventricle (RV) afterload. These factors are pulmonary endothelial dysfunction related to cardiopulmonary bypass (CPB), high sympathetic tone, high MV pressures, pre-existing pulmonary hypertension (PH), and protamine.

In particular, pulmonary endothelial dysfunction occurring during CPB is characterized by a vasoactive mediator imbalance, with reduced levels of nitric oxide (NO) and proteinoids. Additional characteristics include abnormal hypoxic vasoconstriction (80% arteriolar) and micro/macrovascular thrombosis. The result is pulmonary endothelial remodeling with an increased RV afterload and RV dysfunction [5,6].

The most widely shared approach to perioperative RVF management aims to: (1) optimize RV preload; (2) improve RV systolic function with inotropic support, atrioventricular synchronization, or MCS); (3) decrease RV afterload with pulmonary vasodilators, adequate oxygenation (avoiding hypercapnia and acidosis), and minimizing MV (4) maintain adequate right coronary artery perfusion pressure [7].

According to the latest guidelines published by the European Society of Cardiology (ESC) in August 2022, PH is defined by a mean pulmonary artery pressure (mPAP) ≥20 mmHg, while the upper limit of normal PVR is ∼2 Wood units (WU) [8,9,10].

The ESC guidelines highlight that PVR and pulmonary arterial wedge pressure (PAWP) must be used to differentiate pre-capillary PH (due to pulmonary vascular disease) from post-capillary PH (due to left heart disease or elevated pulmonary blood flow).

Several patients with post-capillary PH undergo the phenomenon of pulmonary arterial remodeling and vasoconstriction. This causes an increase in pre-capillary pulmonary resistances leading to a combined pre- and post-capillary PH.

Perioperative alterations in pulmonary vascular tone may dramatically affect RV function. Inhaled pulmonary vasodilators (IPVs) have arisen as a promising treatment in this field. They act as selective pulmonary vasodilators as they are delivered only to ventilated areas. Potential advantages are prevention of perfusion-ventilation mismatching, and reduction of perioperative RV afterload, with no impact on systemic vascular resistances. The present narrative review aims to assess the efficacy of the different IPVs in the treatment of perioperative RVF.

## 2. Inhaled Pulmonary Vasodilators: Mechanisms of Actions and Fields of Application

In 2010, Price LC et al., enhanced the role of IPV in reducing PVR and improving RV function, with a notably better side-effect profile when compared with systemic agents [7].

Three main signaling pathways may be targeted: prostacyclins (PGI2), NO, or endothelin.

Endogenous PGI2 derives from arachidonic acid, which activates the prostacyclin receptor, stimulating cyclic adenosine monophosphate (cAMP). The latter determines both vasodilatation and inhibition of cell proliferation and platelet activation.

The second pathway is NO; this endogenous vasodilator stimulates the release of cyclic guanosine monophosphate (cGMP) from guanylyl cyclase. cGMP has similar effects to cAMP.

The third pathway concerns the endothelin receptor antagonists. The latter decrease the excess of endothelin-1 pathways observed in PH patients [11].

According to current practice standards, a positive acute vasodilator response is defined as a fall in mPAP of at least 10 mmHg, below 40 mmHg, without a reduction in cardiac output. Subjects who experience a 30 mmHg drop in PVR have a better prognosis than non-responders [12].

All IPVs can reduce PVR and improve RV function with minimal systemic effects. This is because they are delivered only to ventilated areas, where their vasodilatory action can enhance blood flow, enhancing ventilation/perfusion matching (Table 1).

### 2.1. Inhaled PGI2

PGI2 was first studied in 1976 by the Nobel Prize winner John Vane. He discovered their potent vasodilatory, antiproliferative, pro-apoptotic, and antithrombotic properties.

The first prostacyclin cleared by the Food and Drug Administration (FDA) for the treatment of PH was Epoprostenol in 1995.

Although Epoprostenol was approved for continuous intravenous infusion, it can be aerosolized and used off-label.

Inhaled Epoprostenol (iEPO) can be used based on patient weight or as a fixed dose.

In cardiopulmonary disease patients, it seems to have similar efficacy to inhaled NO (iNO), but it is much cheaper (i.e., up to a 90% cost drop) [13,14].

Li et al., demonstrated iEPO feasibility via a high-flow nasal cannula (HFNC) and not only via an endotracheal tube [15].

iEPO has some drawbacks such as its very short half-life (3–5 min). Consequently, it requires frequent daily administrations. In addition, it cannot be used as a substitute for the intravenous route because it does not allow medication delivery at high doses.

iEPO has been reported to produce mild acute sterile tracheitis in animal models. However, a recent novel toxicology program showed no drug-related airway or lung inflammation [16].

A synthetic analogue of the endogenous PGI2 is inhaled Iloprost (i-Iloprost). It can be administered over 15 min by a jet nebulizer which adapts to patient breathing patterns.

Its onset of action is 30–60 s with a half-life longer than iEPO (7–8 min). Despite this, it needs to be given at least 6 times/day.

It was approved based on the Aerosolized Iloprost Randomised (AIR) trial, where they tested its effect on the 6 min walk distance during a 12-week treatment [17].

Monotherapy with i-Iloprost has shown significant improvement in the New York Heart Association (NYHA) classification, effort tolerance, and quality of life in PH patients.

It has been claimed to improve RV function and partially reverse RV fibrosis in several studies [18,19], especially in the context of mitral valve operations, coronary artery bypass grafts (CABGs), and heart or lung transplantation [17,20,21].

More specifically, the literature has shown that improvements in RV function were proportionally associated with PVR reduction in idiopathic PH patients, since RVF is secondary to pulmonary hypertension [18].

On the other hand, in connective tissue disorder pulmonary arterial hypertension (CTD-PAH), no significant association was found between PVR and RV function as the damage to the RV comes from direct infiltration of the myocardium. No association between PVR and RV function changes was found in congenital heart disease (CHD) PAH patients treated with i-Iloprost. The potential cause of interference here could be intracardiac shunts [22].

I-Iloprost should be combined with other drugs if used for a prolonged time. If added to Bosentan, i-Iloprost improves hemodynamics, exercise tolerance, and quality of life [23].

It is concerning that some patients in the AIR trial showed serious syncope episodes, mainly during exertion in the morning. Children were intolerant to i-Iloprost because of its irritant airway effects, including coughing and bronchospasm [17].

The third prostacyclin suitable for inhalation is Treprostinil. The latter may be administered in patients with PH who remain symptomatic on Bosentan or Sildenafil, but who are unsuitable for infusion therapy. It is not the first choice for initial therapy because of limited efficacy and high costs. Similar to i-Iloprost, it can be prescribed for outpatients [24].

### 2.2. Inhaled Nitric Oxide (iNO)

iNO was approved by the FDA for the treatment of hypoxic respiratory failure and persistent pulmonary hypertension (PPH) of term and near-term newborns. Its use in adult scenarios is considered off-label [25]. Endogenous NO is released by endothelial cells under acetylcholine stimulation. It is produced by NO synthase (NOS), which combines oxygen with the amino acid L-arginine. It is also produced by macrophages, nerve cells, smooth muscle cells, and epithelial cells. In smooth muscle cells, it promotes the conversion of Guanosine-5’-triphosphate (GTP) to cGMP. The overall effect is a reduction in intracellular calcium along with relaxation of smooth muscle cells and vasodilation [26].

NO is rapidly metabolized to form nitrate and methemoglobin after reaction with oxygenated hemoglobin (Hb). In erythrocytes, methemoglobin reductase converts methemoglobin to ferrous Hb.

Nearly 70% of iNO is excreted as nitrate in the urine within 48 h. It has a half-life of only a few seconds in the serum.

iNO has some potential side effects: surfactant damage, DNA alterations, a risk for methemoglobinemia and systemic dilative effects of its metabolites, and a rebound increase in mPAP after discontinuation [27,28,29,30].

It is not dose-dependent and the maximum effect on the pulmonary vascular system can be achieved at doses as low as 10 ppm. However, toxicity is dose-related [31].

The use of iNO may be controversial. It acts as an anti-proliferative and anti-inflammatory drug in physiologic conditions. In pathologic states, inducible NOS increases NO release, with potential pro-inflammatory and toxic effects, especially when peroxynitrite is formed by the interaction of NO and superoxide. That is why iNO should not be administered together with high fractions of inspired oxygen.

Currently, its use is widespread in several settings during cardiothoracic surgery. It may help to prevent ischemia-reperfusion injury (IRI) after prolonged CPB and reduce vasoconstriction induced by hemolysis. It may provide cardioprotective effects during CPB, reversing or preventing RVF and cardiogenic shock. Finally, iNO can fight hypoxia and PH occurring in thoracic surgery during one-lung ventilation, pneumonectomy, and lung transplantation (LT) [32].

The most specific clinical use of iNO is in the context of LVAD implantation, HT, and LT.

#### 2.2.1. iNO in LVAD

Patients with end-stage HF due to severe left ventricular (LV) systolic dysfunction usually suffer from PH. PH might be the sequel to high LV filling pressure, reactive pulmonary vasoconstriction, and chronic pulmonary vascular remodeling. Treatment of HF with LVAD may passively reduce PVR by decreasing the PAWP.

However, up to 40% of LVAD recipients may experience post-LVAD RVF due to: (1) increased venous return; (2) increased blood flow through the pulmonary vessels; (3) acute pulmonary vasoconstriction due to CPB and blood product transfusion; and (4) changes in RV geometry due to the left-forward shift of the interventricular septum.

RVF and PH may in turn reduce LVAD preload, leading to decreased organ perfusion. Treatment with inotropes and intravenous pulmonary vasodilators is often complicated by arrhythmias and hypotension [33,34].

When the PVR is >3 Wood units, or TPG is >12 mmHg, iNO is the preferred selective pulmonary vasodilator, resulting in decreased PVR, an increased RV ejection fraction, and improved LVAD performance [35].

Despite this, a randomized controlled trial on 150 patients with elevated PVR undergoing LVAD implantation reported no significant reduction in RVF incidence in patients treated with iNO at 40 ppm for 48 h when compared with the placebo [36].

#### 2.2.2. iNO in Lung Transplantation

Although iNO seems to be an effective tool in patients undergoing LVAD implantation, its use in patients receiving LT is still controversial.

The prophylactic institution of iNO has shown a reduced incidence of ischemia-reperfusion injury of the lung [37]. However, three more recent randomized clinical trials showed that iNO after LT had no significant effect on oxygenation or prevention of primary graft dysfunction (PGD) [38,39,40].

#### 2.2.3. iNO in Heart Transplantation

iNO is a good option during right heart catheterization in heart transplantation candidates as it helps to identify patients with reversible PH. In the most recent American Heart Association (AHA) and American Thoracic Society (ATS) guidelines, “iNO or iPGI2 should be used as the initial therapy for pulmonary hypertensive crisis and RVF (Class I: Level of Evidence B)” after cardiac surgery, although they do not mention any evidence or recommendations for the prophylactic use of iNO after a heart transplant [41].

In a meta-analysis conducted by Rea et al., iNO shows early hemodynamic benefits in heart transplant patients with pre-existing pulmonary hypertension, and variable hemodynamic benefits in lung transplant recipients. Currently, morbidity and mortality data are not favorable for either indication [42].

Further powered studies are needed to define the effect, dose, and timing of iNO in heart transplantation patients.

## 3. Inhaled Milrinone

Inhaled milrinone is not currently FDA-approved [43].

Recent data suggest its use for acute RVF. However, absorption from the pulmonary circulation can lead to systemic hypotension and cardiac arrhythmias [44].

## 4. Inhaled Sildenafil

Oral sildenafil was approved by the FDA in 2007 for Group 1 PAH. It has relative pulmonary specificity. The inhaled formula has been tested in animal models with no significant effects on systemic blood pressure [45].

## 5. Inhaled Levosimendan

Levosimendan is a calcium sensitizer with inotropic and vasodilating properties.

Only a single study has claimed the same effectiveness as milrinone in reducing mPAP, with a longer duration of action. Larger randomized clinical trials are needed to support its use [46].

## 6. INO vs. iPGI2: Which One to Use?

### 6.1. I-Iloprost vs. iNO

Most studies support the efficacy of combination therapy (I-Iloprost + iNO) in LVAD patients, with a statistically significant decrease in the requirement for a right ventricular assist device (Table 2) [47].

Combination therapy eliminates the risk of rebound increase in PVR and mPAP after iNO discontinuation. I-Iloprost is suitable for further administrations after the patient is extubated [54,55,56].

### 6.2. iEPO vs. iNO

iEPO and iNO showed similar efficacy in reducing mPAP immediately after cardiac surgery and no difference in the rate of bleeding or systemic hypotension has been found.

Both agents were shown to reduce PVR by 20% in non-surgical PAH patients. Nevertheless, no additive effects (beneficial or detrimental) have been observed when delivered in combination [48].

iNO and i-EPO have shown similar efficacy in terms of mPAP reduction and P/F ratio improvement in the context of HT and LT. No difference after crossover has been found.

Potential advantages of using iEPO vs. iNO include eliminating the risk of methemoglobinemia, easier administration, and cost savings [49,50,51,57].

A clinical investigation (INSPIRE-FLO) with an aim to compare i-iEPO and iNO in adult patients undergoing LVAD placement, HT, or LT is still ongoing. Primary outcomes of the investigation are the incidence of Grade 3 PGD in LT subjects (up to 72 h) and the incidence of moderate to severe RVF in LVAD and HT patients.

Preliminary results, updated on the 16th of February 2023, report that the incidence of PGD is 39.8% in patients receiving iNO vs. 44.7% in patients receiving iEPO (*p*-value 0.019). In addition, the incidence of severe RVF after LVAD implantation or HT is 22.5% in the iNO group vs. 25% in the iEPO group (*p*-value 0.012) [52,53].

Interestingly, in vitro studies on myocardial tissue preparations showed how the increase in RV function after iPG12 could not be fully explained by a decreased afterload. It can be explained by a direct prostacyclin-induced positive inotropism [58,59,60].

However, in most studies, the effects of prostacyclin on cardiac output (CO) have been reported to be variable with no changes, decreases, or, more often, increases in contractility [61,62,63].

## 7. Discussion and Conclusions

The use of IPV has been the subject of great interest in the last few years. They are widely accepted for the treatment of perioperative RVF because of the temporary alterations in pulmonary vascular tone occurring during cardio-thoracic surgery (HT, LT, or LVAD implantation). Apart from the evidence of clinical similarity between iNO and iPGI2, the cost-saving difference remains a problem and is one of the main topics in the latest editorials and original articles [64,65].

Several concerns about the use of IPV do exist.

First, they should be administered with caution in patients with PH caused by venous-occlusive diseases or left heart failure, due to the increase in pulmonary venous congestion and pulmonary capillary wedge pressure [66,67].

Furthermore, strong scientific evidence supporting their pre-emptive use in the context of LVAD, HT, and LT is still poor [32,68,69].

More specifically, the already published clinical trials failed to demonstrate the benefit of iNO for the prevention of RVF in patients undergoing LVAD implantation and for the prevention of PGD by early administration during LT.

We are waiting for the final analysis of INSPIRE-FLO (last update Feb 2023) [52,53].

To our knowledge, this current study is the largest blind single-center randomized controlled trial on adult patients addressing whether iEPO is clinically equivalent to iNO after HT and LVAD placement in preventing RVF, and whether they are equivalent in preventing PDG after LT. 

In HT recipients, RVF has been defined by the need for an MCS device (RV-assist device [RVAD] or venoarterial extracorporeal membrane oxygenation) for isolated RVF within 30 days of surgery.

For LVAD patients, RVF has been defined by moderate to severe right heart failure criteria according to INTERMACS [70,71].

The INSPIRE-FLO investigation has found a risk difference of 2.5% between the iEPO and iNO groups and sufficient evidence to demonstrate that iNO and iEPO are similar in the prevention of postoperative RVF. No significant between-group differences have been observed in the duration of mechanical ventilation, ICU and hospital length of stay, tracheostomy placement, renal replacement therapy initiation, or mortality up to 1 year after surgery.

For LT, they have considered the incidence of PGD as the primary outcome. They have found a risk difference of 4.9% in support of equivalence between the two pre-emptive treatments.

The INSPIRE-FLO investigation confirms what the already published studies, although limited, have shown: the effects of iNO and iEPO are similar in a mixed population of cardiac surgical patients.

The results of this relevant investigation could even change the clinical routine.

Future directions should include the implementation of larger multicenter trials to strengthen these findings.

In current clinical practice, iNO is used mainly as a diagnostic agent or for short-term acute rescue therapy, although it has not been approved by the FDA for this scenario.

I-Iloprost and inhaled Treprostinil are mainly used in patients already on one or two background therapies who have not achieved the therapeutic goals, but who have not deteriorated enough to require an infusion prostacyclin therapy [72].

Considering everything mentioned above, we may conclude that:

- when RVF occurs in the setting of normal PVR, inotropic therapy should be sufficient to improve RV output.
- when RVF occurs in the setting of elevated PVR or the patient has evidence of a high RV afterload (TPG > 12 mm Hg), inhaled pulmonary vasodilators would be the preferred initial agents. The literature seems to support their pre-emptive use in patients at high risk of developing postoperative RVF.
- If severe refractory postoperative PH and overt RVF occur, IPV should be combined with complementary pharmacology (inotropes and inodilators) as salvage therapy. If unsuccessful, RV mechanical support should be established.

## Figures and Tables

**Table 1:** Inhaled pulmonary vasodilators.

Inhaled Pulmonary Vasodilator	Indications	Administration	Dose	Onset of Action	Half Life	Cost	Outcomes	Adverse Effects
I–Epoprostenol	Vasoreactivity testing * Hypertensive crisis *	Aerosolized solution	50 g/via mask for 10 min or hours to day	30–60 s	3-5 min	$36/ administration	Decreased mPAP Decreased PVR Increased oxygenation	Withdrawal Headache Jawache Nausea Diarrhea
I–Iloprost	Group 1 PAH Improves exercise tolerance and avoids deterioration	Aerosolized solution	2.5 or 5 g/dose 6–9 times/day or continuous nebulisation	30–60 s	30 min	$70,000/year	Decreased mPAP Increased 6MWT tolerance	Cough Wheeze
I–Treprostinil	Group 1 PAH Improve exercise tolerance and avoids deterioration	Puffs	3–9 puffs 4 times/die	60 min	3–4 h	$100,000/years	Increased 6MWT tolerance	Cough Headache Sorethroat Irritation Nausea Diarrhea Syncope
i-Nitrix Oxide	PPHN Vasoreactivity testing * Hypertensive crisis *	Gas	5–40 ppm for hours to days	5–10 s	10–20 s	$100–400/h	Decreased mPAP Decreased PVR Improved oxygenation Increased 6 MWT	Possible withdrawal Increased methemoglobin

Abbreviations: mPAP, mean pulmonary arterial pressure; PAH, pulmonary arterial hypertension; PPHN, persistent pulmonary hypertension of the newborn; PVR, pulmonary vascular resistance; 6MWT, 6 min walking test. * Off-label indications.

**Table 2:** Studies comparing iNO to iPGI2 in cardiac surgical and non-surgical patients with pulmonary hypertension.

Authors	Year	Type of Study	Patients’ Population	Group	Administration Dose	Timing	Size	Overall Results
Winterhalter et al. [47]	2008	Prospective, randomized, single centre	Cardiac surgery (HT and LVAD excluded)	iNO	20 ppm	immediately after weaning from CPB	23	i-Iloprost more effective in decrease PVR, mPAP and increase CO
				i-iloprost	20 mcg/2 mL (aerosolized)	4–6 min after weaning from CPB	22	
Preston et al. [48]	2013	Prospective, randomized, single centre	IPH and HFpEF patients	IPH (PAWP < 15 mmHg)	iNO 20 ppm and iEPO 50 ng/kg/min	during right catheterization *	12	Exposure of HFpEF patients to inhaled vasodilators worsens the PAWP without hemodynamic benefit
				HFpEF (PAWP 16–25 mmHg)	iNO 20 ppm and iEPO 50 ng/kg/min		7	
Khan A et al. [49]	2009	Prospective, randomized, single centre	HT and LT	iNO	20 ppm	immediately after weaning from CPB if pulmonary PH, refractory hypoxemia, or RVF	14	No differences in decrease mPAP, PVC or increase CI or venous oxygen saturation
				iEPO	20 ng/mL 8 mL/h		11	
McGinn et al. [50]	2016	Retrospective, single-centre, observational	Cardiac surgery **	iNO	10–40 ppm	after weaning from CPB if acute PH	49	No difference in decrease mPAP, significant cost saving with iEPO
				iEPO	20 ng/mL 8–12 mL/h		49	
Fattouch et al. [51]	2005	Prospective randomized, single center, double-blind	Mitral valve surgery (MVS with elevated PVR)	iNO	20 ppm	immediately after admission in ICU	22	No difference in decreased mPAP, PVR and increased CO. PGI2 free from toxic effect and easier to administer
				iPGI2	10 ng/mL rates of 0.3 mL/h		18	
Ghadimi et al. [52] for the INSPIRE FLOW Investigation	2023	Prospective randomized duoble blind, single center	HT and LVAD	iNO	20 ppm	15 min before weaning from CPB	111	No difference in RVF development
				iEPO	50 ng/kg/m		120	
Ghadimi et al. [53] for the INPIRE FLOW Investigation	2023	Prospective randomized duoble blind, single center	LT	iNO	20 ppm	15 min before reperfusion of the first lung	108	No difference in PGD development
				iEPO	50 ng/kg/min		112	

Abbreviations: CI, cardiac index; CO, cardiac output; CPB, cardiopulmonary bypass; HFpEF, heart failure with preserved ejection fraction; HT, heart transplant; ICU, intensive care unit; iEPO, inhaled epoprostenol; i-Iloprost, inhaled iloprost; iNO, inhaled nitric oxide; iPGI2 inhaled prostacyclins; IPH, idiopathic pulmonary hypertension; LT, lung transplant; LVAD, left ventricular assist device; mPAP, mean pulmonary arterial pressure; MVS, mitral valve stenosis; PAWP, pulmonary arterial wedge pressure; PH, pulmonary hypertension; PVR, pulmonary vascular resistances; RVF, right ventricular failure. * Preston et al. [48]. Hemodynamic parameters were measured at baseline 15 min after the administration of each vasodilator, and after 20 min of a washout phase prior to the administration of the next agent to ensure the return of hemodynamics to baseline. ** McGinn et al. [50]. Hemodynamic and oxygenation parameters were recorded before and after initiation of pulmonary vasodilator therapy.At 6 h, the hemodynamic and oxygenation parameters were recorded again, just before discontinuing the initial agent. Crossover baseline parameters were measured 30 min after the initial agent had been stopped. The crossover agent was then started, and the hemodynamic and oxygenation parameters were measured again 30 min later.
